# Immobilization of *C. elegans* with different concentrations of an anesthetic for time-lapse imaging of dynamic protein trafficking in neurons

**DOI:** 10.17912/micropub.biology.001069

**Published:** 2024-01-16

**Authors:** Cesar Siete, Rui Xiong, Anaam Khalid, Yi-Wen Hsieh, Chiou-Fen Chuang

**Affiliations:** 1 Department of Biological Sciences, University of Illinois at Chicago, Chicago, Illinois, United States; 2 Graduate Program in Neuroscience, University of Illinois at Chicago, Chicago, Illinois, United States

## Abstract

Here we compare the percentage of anterograde and retrograde trafficking events as well as the average velocity of these events in worms immobilized with microbeads or 0.5-7.5 mM tetramisole. Our results show that the percentage and average velocity of TIR-1 ::GFP moving events in the *C. elegans* AWC axons are not significantly different between worms immobilized with 7.5 mM tetramisole and other conditions. Our results suggest that 7.5 mM tetramisole, compared to 0.5 mM, 1 mM, and 2 mM tetramisole, does not have a significant effect on the axonal transport of TIR-1 ::GFP along the AWC axons.

**Figure 1. Comparison of axonal transport of TIR-1::GFP in AWC axons of worms immobilized with different concentrations of tetramisole and polystyrene microbeads:**
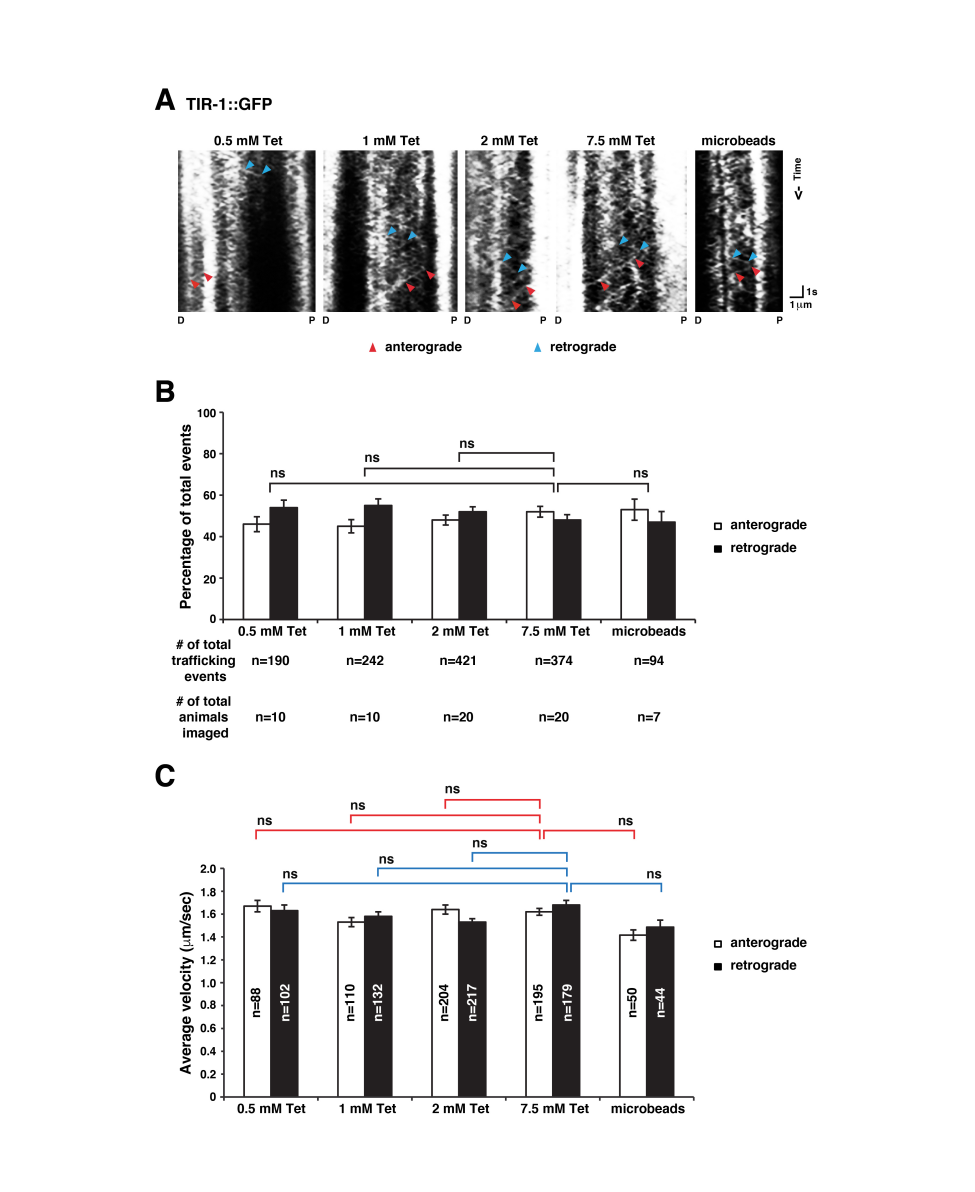
( **A** ) Representative kymographs of moving TIR-1::GFP in AWC axons of worms immobilized with 0.5-7.5 mM tetramisole (Tet) or microbeads. The line within two blue arrowheads represents a retrograde trafficking event toward the proximal axon (P) or the ­­­­­AWC cell body; the line between two red arrowheads represents an anterograde trafficking event toward the distal (D) axon. ( **B** ) The percentage of anterograde and retrograde trafficking events. The average number of trafficking events per animal imaged is 19 (0.5 mM Tet), 24 (1 mM Tet), 21 (2 mM Tet), 19 (7.5 mM Tet), and 13 (microbeads). *Z* -test was used to determine *p* -values. Error bars, SE of proportion. ns, not significant. ( **C** ) The average velocity of anterograde and retrograde trafficking events. Red and blue lines indicate the comparison of anterograde and retrograde velocities, respectively. Student’s *t-test* was used to determine *p* -values. Error bars, SEM. ns, not significant. The significance level 0.01 was adjusted to 0.0025 (0.01/4) using the Bonferroni method (Chen, Feng, & Yi, 2017) in (B) and (C).

## Description

To perform dynamic imaging of protein trafficking events or mitochondrial transport in *Caenorhabditis elegans* , worms are usually immobilized with anesthetics (Mondal, Ahlawat, Rau, Venkataraman, & Koushika, 2011) or microfluidics (Mondal et al., 2011; Mondal et al., 2021) . Polystyrene microbeads (Kim, Sun, Gabel, & Fang-Yen, 2013) and glue (Kerr et al., 2000) are potentially good alternatives to conventional immobilization of worms for time-lapse imaging of protein/organelle moving events in neurons. Cholinergic agonists, such as tetramisole and levamisole that cause hypercontracted paralysis by mediating excitatory neurotransmission at the neuromuscular junctions, are commonly used for time-lapse imaging in *C. elegans* . Mitochondrial respiratory chain inhibitors like sodium azide, another common anesthetic used for immobilization of *C. elegans* , block metabolic activity of the mitochondria and thus are not suitable for time-lapse imaging. As tetramisole and levamisole act on acetylcholine receptors, it has long been a concern that they may affect subcellular processes (Mondal et al., 2011) . Despite this, tetramisole and levamisole have still been used to image the axonal transport of proteins or mitochondria in *C. elegans* as seen in recent studies (Barmaver et al., 2022; Eichel et al., 2022; Vasudevan et al., 2023) .

Considering logically, lower concentrations of anesthetics should have less effect on subcellular processes, but higher concentrations should better immobilize animals. With this idea in mind, we wanted to determine whether different concentrations of tetramisole would actually affect the dynamic movement of the TIR-1 (Sarm1) scaffold protein that we have been studying in the asymmetric differentiation of the *C. elegans* AWC olfactory neuron pair (Chang, Hsieh, Lesch, Bargmann, & Chuang, 2011; Chuang & Bargmann, 2005) . In a previous study, we used 6mM levamisole (Chang et al., 2011) to immobilize worms for dynamic imaging of TIR-1 ::GFP in AWC axons. In this study, we compared the percentage and velocity of anterograde and retrograde trafficking events of TIR-1::GFP in worms immobilized with 0.5 mM, 1 mM, 2 mM tetramisole, or microbeads (without the use of anesthetics) against 7.5 mM tetramisole. Although another study has compared various concentrations of different anesthetics with microfluidic devices to immobilize animals for time-lapse imaging of several cellular and subcellular processes (Mondal et al., 2011) , our study was specifically focused on the effect of tetramisole concentrations on the axonal transport of TIR-1 ::GFP in AWC neurons.

We took time-lapse images of an integrated *odr-3p:: tir-1 ::GFP* transgene, which expressed functional TIR-1 ::GFP fusion protein in AWC neurons, to visualize TIR-1::GFP movement in AWC axons *.* We then compared anterograde and retrograde events between different concentrations of tetramisole (0.5 mM, 1 mM, 2 mM, or 7.5 mM) and microbeads after creating kymographs ( Figure 1A ) to track the direction and velocity of protein movement. We analyzed 94-421 moving events obtained by imaging 7-20 animals immobilized with different conditions ( Figure 1B and 1C). Bonferroni adjustment (Chen, Feng, & Yi, 2017) was used for multiple comparisons ( Figure 1B and 1C). We found no significant differences in the percentage of anterograde and retrograde events of worms immobilized with different concentrations (0.5 mM, 1 mM, or 2 mM) of tetramisole or microbeads, compared to 7.5 mM tetramisole-immobilized worms ( Figure 1B ). In addition, we found no significant difference in either anterograde or retrograde velocity of TIR-1 ::GFP movement between worms immobilized with 7.5 mM tetramisole and other conditions ( Figure 1C ). However, we found morphological changes and bursting of worms within minutes of mounting with microbeads, likely due to our limited familiarity and experience using polystyrene microbeads. Taken together, our results support that using 7.5 mM tetramisole, compared to lower concentrations (0.5 mM, 1 mM, and 2 mM) of tetramisole, to immobilize worms for­­­­ dynamic imaging has no significant effect on the transport of TIR-1::GFP along the AWC axons.

## Methods

**Time-lapse imaging of protein trafficking**

Worms in the L2 larval stage were anesthetized with 0.5 mM, 1 mM, 2 mM, or 7.5 mM tetramisole and mounted onto 2% agarose pads on microscope slides for imaging; or immobilized with polystyrene microbeads and mounted onto 5% agarose pads on microscope slides for imaging. Time-lapse images were acquired for 30 seconds with a speed of 5 frames per second and an exposure time of 200 milliseconds using a Zeiss Axio Imager M2 microscope, equipped with a Zeiss objective EC Plan-Neofluar 63x/1.40 Oil DIC M27, a Piston GFP bandpass filter set (41025, Chroma Technology), a Hamamatsu digital camera C11440, and the Zeiss imaging software ZEN (2012 blue edition SP2). Acquired images were analyzed to generate kymographs using the Fiji software (Schindelin et al., 2012) with the KymographClear macro (version 2.0a) (Mangeol, Prevo, & Peterman, 2016) . The percentage and velocity of moving events were measured using the KymographDirect software (version 2.1) (Mangeol et al., 2016) .

## Reagents

**Table:** 

Strain	Genotype	Source
IX2738	*vyIs62* [ *odr-3p:: tir-1 ::GFP* (10 ng/μl) *; ofm-1p::DsRed* (30­ ng/μl)] X	This study

**Table:** 

Chemical	Supplier
tetramisole	Sigma, catalog number: L9756
